# Prevalence of self-medication in Brazil and associated factors

**DOI:** 10.1590/S1518-8787.2016050006117

**Published:** 2016-12-01

**Authors:** Paulo Sérgio Dourado Arrais, Maria Eneida Porto Fernandes, Tatiane da Silva Dal Pizzol, Luiz Roberto Ramos, Sotero Serrate Mengue, Vera Lucia Luiza, Noemia Urruth Leão Tavares, Mareni Rocha Farias, Maria Auxiliadora Oliveira, Andréa Dâmaso Bertoldi

**Affiliations:** I Departamento de Farmácia. Faculdade de Farmácia, Odontologia e Enfermagem. Universidade Federal do Ceará. Fortaleza, CE, Brasil; II Departamento de Produção e Controle de Medicamentos. Faculdade de Farmácia. Universidade Federal do Rio Grande do Sul. Porto Alegre, RS, Brasil; III Departamento de Medicina Preventiva. Escola Paulista de Medicina. Universidade Federal de São Paulo. São Paulo, SP, Brasil; IV Departamento de Medicina Social. Universidade Federal do Rio Grande do Sul. Porto Alegre, RS, Brasil; V Departamento de Política de Medicamentos e Assistência Farmacêutica. Escola Nacional de Saúde Pública Sérgio Arouca. Fundação Oswaldo Cruz. Rio de Janeiro, RJ, Brasil; VI Departamento de Farmácia. Faculdade de Ciências da Saúde. Universidade de Brasília. Brasília, DF, Brasil; VII Departamento de Ciências Farmacêuticas. Centro de Ciências da Saúde. Universidade Federal de Santa Catarina. Florianópolis, SC, Brasil; VIII Departamento de Medicina Social. Faculdade de Medicina. Universidade Federal de Pelotas. Pelotas, RS, Brasil

**Keywords:** Self Medication, Drug Utilization, Socioeconomic Factors, Pharmacoepidemiology, Health Surveys

## Abstract

**OBJECTIVE:**

To analyze the prevalence and associated factors regarding the use of medicines by self-medication in Brazil.

**METHODS:**

This cross-sectional population-based study was conducted using data from the PNAUM (National Survey on Access, Use and Promotion of Rational Use of Medicines), collected between September 2013 and February 2014 by interviews at the homes of the respondents. All people who reported using any medicines not prescribed by a doctor or dentist were classified as self-medication practitioners. Crude and adjusted prevalence ratios (Poisson regression) and their respective 95% confidence intervals were calculated in order to investigate the factors associated with the use of self-medication by medicines. The independent variables were: sociodemographic characteristics, health conditions and access to and use of health services. In addition, the most commonly consumed medicines by self-medication were individually identified.

**RESULTS:**

The self-medication prevalence in Brazil was 16.1% (95%CI 15.0–17.5), with it being highest in the Northeast region (23.8%; 95%CI 21.6–26.2). Following the adjusted analysis, self-medication was observed to be associated with females, inhabitants from the North, Northeast and Midwest regions and individuals that have had one, or two or more chronic diseases. Analgesics and muscle relaxants were the therapeutic groups most used for self-medication, with dipyrone being the most consumed medicines. In general, most of the medicines used for self-medication were classified as non-prescriptive (65.5%).

**CONCLUSIONS:**

Self-medication is common practice in Brazil and mainly involves the use of non-prescription medicines; therefore, the users of such should be made aware of the possible risks.

## RESUMO

**OBJETIVO:**

Analisar a prevalência e os fatores associados à utilização de medicamentos por automedicação no Brasil.

**MÉTODOS:**

Este estudo transversal de base populacional foi realizado com dados da Pesquisa Nacional de Acesso, Utilização e Promoção do Uso Racional de medicamentos (PNAUM), coletados de setembro de 2013 a fevereiro de 2014, por meio de entrevistas em domicílio. Todas as pessoas que referiram usar qualquer medicamento sem prescrição por médico ou dentista foram classificadas como praticantes de automedicação. Foram calculadas razões de prevalência bruta e ajustada (regressão de Poisson) e seus respectivos intervalos de confiança de 95% na investigação dos fatores associados ao consumo de medicamentos por automedicação. As variáveis independentes foram: aspectos sociodemográficos, de condições de saúde e de acesso e utilização de serviços de saúde. Adicionalmente, foram identificados os medicamentos mais consumidos por automedicação.

**RESULTADOS:**

A prevalência da automedicação no Brasil foi de 16,1% (IC95% 15,0–17,5), sendo maior na região Nordeste (23,8%; IC95% 21,6–26,2). Após análise ajustada, automedicação mostrou-se associada a ser do sexo feminino, pertencer às faixas etárias 10-19 anos, 20-29 anos, 40-59 anos e 60 anos ou mais, residir na região Norte, Nordeste ou Centro-Oeste, e ter uma ou duas ou mais doenças crônicas. Os analgésicos e os relaxantes musculares foram os grupos terapêuticos mais utilizados por automedicação, sendo a dipirona o fármaco mais consumido. No geral, a maioria dos medicamentos usados por automedicação foram classificados como isentos de prescrição (65,5%).

**CONCLUSÕES:**

A automedicação é prática corrente no Brasil e envolve, principalmente, o uso de medicamentos isentos de prescrição, devendo os usuários ficarem atentos aos seus possíveis riscos.

## INTRODUCTION

Medications are important social goods that are heavily used by the Brazilian population^4^. The use of these drugs is influenced by various factors. Some of these factors are in relation to the Brazilian population’s increase in life expectancy, the consequent growing frequency of chronic disease, the emergence of new and old transmittable diseases, the increasing prevalence of mood disorders, diseases arising from environmental degradation, environmental pollution and climate change, and the growing financial investment made by the Brazilian government to ensure access to health care services for all^17^.

There are still difficulties regarding access, delay and low quality of care in health services, both in the public and private sectors, all of which exist despite the advances made. In addition to these aspects, the marketing of non-prescription drugs in the media, the existence of home-stored medication and the belief that drugs solve all health problems, are important factors involving self-medication^16^.

The World Health Organization^a^ (1998) defines self-medication as the selection and use of non-prescription drugs without supervision from a physician or dentist. Self-medication is a worldwide phenomenon, the prevalence of such can differ depending on the population studied, the method and the recall period employed: in Germany^9^, the prevalence for medication use on a self-medication basis was 27.7%; in Portugal^14^, this was 26.2%; in Spain^7^, 12.7%; in Cuba^8^, 7.3%; in Athens-Greece^2^, 23.4%; in the Catalonia region of Spain^20^, 34.0% among men and 25.0% among women; and in Pondicherry-India^22^, this was equal to 11.9%.

There have been few population-based studies in Brazil to have investigated the pattern of medication consumption for the Brazilian population as a whole^5^ ^,^ ^6^. A study performed by Carvalho et al.^4^ (2005) showed that overall prevalence of medication use by the section of the population older than 18 years, 15 days before interview, was 49.0% and 24.6% for self-medication. Other studies focus on the populations from Brazilian municipalities. The prevalence of self-medication ranged from 27.0% and 32.0% among residents, aged over 40 years^21^, from the city of Sao Paulo (Sao Paulo state); whereas the prevalence of exclusive consumption of non-prescription drugs was 28.8% in Bambui, MG, Southeastern Brazil, a result found during a study with people aged 18 years or over^12^. In Santa Maria, RS, Southern Brazil, 76.1% of the interviewed individuals claimed to have self-medicated at least once^24^.

In this context, considering the small number of published studies that have a national representation, the PNAUM (National Survey on Access, Use and Promotion of Rational Use of Medicines), developed by the Brazilian Ministry of Health, makes it possible to evaluate the self-medication situation in Brazil as a relevant theme, given the inherent risks to its practice (medicinal poisoning and adverse reactions) and the possible increase in health care spending^13^ ^,^ ^18^ ^,^ ^19^.

Therefore, the objective of this study was to analyze the prevalence and factors associated with the use of drugs by self-medication in Brazil.

## METHODS

Data from PNAUM were used in this population-based cross-sectional study. The survey was conducted in Brazil, between September 2013 and February 2014 using probabilistic samples randomly drawn from eight demographic aspects according to age group and gender, the individuals involved in the study were residing in the urban areas from each Brazilian region, including the capitals, with sampling by conglomerates in three stages. During the first stage, the municipalities were drawn (n = 60 per region); the second, the census sectors (two in each municipality); and the third, the households. 41,433 individuals were interviewed in total. Further information regarding the sampling plan and the size of the sample are described in PNAUM’s methodological article^15^.

The structured questionnaire was created and tested beforehand by a team of researchers from PNAUM. The interviews were conducted in the selected households by trained researchers, who used electronic equipment (tablets) in order to collect the data.

Information relating to those below 15 years of age and the disabled were provided by their legal guardians (proxies). The questionnaire was modified for this population subgroup, with some blocks of questions that required the respondent’s judgment being excluded (see methodological article^15^).

The outcome of the study is the use of drugs for self-medication. Information regarding medication consumption was obtained in three ways: first, with direct questions related to the use of medicines that had been medically indicated to treat highly prevalent chronic diseases (hypertension; diabetes; heart disease; high cholesterol; stroke; chronic lung disease; arthritis, osteoarthritis or rheumatism; depression) and other diseases that had lasted more than six months at the time of the interview. The second, by a question regarding the use of any medications to treat acute diseases (infection; medicine for sleep or to control nervous conditions; stomach or intestinal problems; fever; pain; influenza; cold or allergic rhinitis; for nausea and vomiting or other acute problem), during the 15 days prior to the interview. The third was in regards to the use of contraceptives (oral and injectable), at the time of the interview. In the second and third methods involved asking who recommended the medicine (doctor or dentist; nurse; pharmacist; another health professional; self-employed; relative, friend or neighbor; spouse or common law partner; partner or boyfriend/girlfriend; pharmacy clerk; other). Thus, all those who reported consuming at least one medication without medical or dentist prescription, during the 15 days prior to the interview, including contraceptives, were classified as self-medicating practitioners.

The interviewers asked all the respondents to submit to them the drugs that they continually used or those they used in the previous 15 days, at the time of the interview. The medicines were classified according to the Anatomical Therapeutic Chemical Classification System, namely the ATC classification system (level 1, organs or systems; level 2, therapeutic subgroup; level 5, drug)^25^ and, according to the legal category, in non-prescription drugs (NPD)^b^, for sale under prescription and medicine controlled^c^.

The exploratory variables were as follows: sociodemographic characteristics, descriptors for health condition, access and use of health services. The sociodemographic variables were: gender; age in years; skin color (white, black, yellow, brown, indigenous); Region of Brazil (North, Northeast, Midwest, South, Southeast); Education in years (0-8 years, 9-11, 12 or more); economic classification (A/B, C, D/E), according to Brazilian Economic Classification Criterion of the Brazilian Association of Research Companies (CCEB 2013/ABEP – http://www.abep.org). The descriptors for health condition were: number of chronic illnesses as reported by the individual (none; one; two or more). The descriptor for access to health services was: health insurance (yes; no). The descriptor for use of health services was: hospitalization in the 12 months prior to the interview (yes; no). This study resulted in a distribution being found regarding the prevalence of self-medication in Brazil’s five regions, which were according to the sociodemographic aspects.

An exploratory descriptive analysis was conducted on all the variables involved in the study and a presentation of the relative frequencies and their respective 95% confidence intervals (95%CI). The Poisson regression model was used to estimate the crude and adjusted prevalence ratios (PR), and the 95%CI. These analyses were conducted with Stata version 12 statistical software, using the set of *svy* commands designed to analyze complex samples and ensure the necessary weighting, with the sampling design being a matter for consideration. Variables with p < 0.20 were included in multiple model and a significance level of 5% was adopted to maintain the variables in the model, along with backward selection of the variables. The statistical significance of the prevalence ratios obtained from the Poisson regression models was evaluated using the Wald test.

This study was conducted in accordance with the recommendations set out in the Declaration of Helsinki and Brazilian Legislation for Ethics in Research. The study was approved by the Brazilian National Commission for Ethics in Research (Protocol 398,131, of 16 September, 2013).

## RESULTS

Six hundred of the 41,433 people interviewed were excluded for not providing information regarding who recommended the drug they were using.

The prevalence of self-medication in the Brazilian population was 16.1% (95%CI 15.0–17.5); the prevalence was higher in female individuals, those aged between 20-39 years, among individuals who declared themselves to be of indigenous and yellow skin color, with time spent in education equal to or greater than 12 years, residents of the Northeast region, between the one and two or more chronic illnesses and those who were hospitalized one or more times in the last year (Table 1).

**Table 1:** Prevalence for the use of at least one drug by self-medication, according to demographic, socioeconomic aspects, health conditions indicators and access to health services indicators. PNAUM, Brazil, 2014. (N = 40,833)

Variable	Prevalence (%)^a^	95%CI	p
Gender			< 0.001
Female	19.0	17.4–20.5	
Male	13.1	11.7–14.6	
Age group (years)			< 0.001
0-9	6.6	5.5–8.0	
10-19	12.6	10.7–14.7	
20-39	21.6	19.6–23.7	
40-59	17.7	16.2–19.4	
≥ 60	14.3	12.9–15.8	
Color			< 0.001
White	15.4	14.1–16.9	
Black	18.4	16.0–21.0	
Yellow	25.2	19.1–32.6	
Brown	17.2	15.5–19.1	
Indigenous	29.8	17.6–45.7	
Education (years)			< 0.001
0-8	14.7	13.4–16.0	
9-11	19.0	17.1–21.0	
≥ 12	19.4	16.8–22.3	
Economic classification^b^			0.866
A/B	16.3	14.4–18.5	
C	16.3	14.8–17.8	
D/E	15.8	14.1–17.7	
Region			< 0.001
North	17.8	15.0–21.0	
Northeast	23.8	21.6–26.2	
Southeast	12.8	10.9–14.9	
South	11.4	10.1–12.9	
Midwest	19.2	17.3–21.3	
No. chronic diseases			< 0.001
0	14.6	13.3–15.9	
1	18.8	16.8–21.1	
2 +	20.4	18.6–22.3	
Health insurance			0.709
Yes	16.5	14.5–18.7	
No	16.1	14.7–17.5	
Hospitalized in the previous year			< 0.001
Yes	20.6	18.2–23.3	
No	16.0	14.7–17.3	
Total	16.7	15.4–18.0	

^a^ Percentages adjusted for sample weights and post-stratified according to age and gender.

^b^ The Economic Classification variable is according to the 2013 Brazilian Economic Classification Criterion of the Brazilian Association of Research Companies (www.abep.org).

In regards to the distribution of self-medication prevalence, according to the sociodemographic aspects, based on the five regions in Brazil (Table 2), women and people in the 20 to 39 years age group consumed a larger quantity of self-medication drugs in all regions of the country, when compared with men. In both situations, the highest prevalences were in the Northeast and the Midwest regions. The prevalence increased gradually from the 0-9 years to the 20-39 years age group, and subsequently declined in all the regions. The prevalence also increased along with time spent in education and with better socioeconomic level, the exception being the North region.

**Table 2:** Prevalence for the use of at least one drug by self-medication, according to demographic and socioeconomic aspects by geographic region. PNAUM, Brazil, 2014a. (N = 40,833)

Variable	Self-medication prevalence									
	North		Northeast		Southeast		South		Midwest	
	%	95%CI	%	95%CI	%	95%CI	%	95%CI	%	95%CI
Gender										
Female	19.2	15.9–23.0	27.5	25.1–30.0	15.3	13.0–18.0	13.1	11.6–14.8	22.9	20.3–25.6
Male	16.3	13.4–19.6	19.6	16.9–22.6	9.9	7.8–12.4	9.5	8.1–11.2	15.5	13.1–18.4
Age group (years)										
0-9	10.9	8.1–14.5	11.9	9.2–15.2	2.5	1.4–4.3	3.6	2.3–5.6	8.7	6.5–11.5
10-19	15.5	10.8–21.7	19.6	15.6–24.3	9.3	6.7–12.8	5.6	3.7–8.2	17.0	12.6–22.7
20-39	23.1	19.5–27.0	31.2	27.5–35.2	17.4	14.4–20.9	15.1	12.8–17.8	24.3	20.9–28.0
40-59	18.9	15.4–22.9	25.6	23.1–28.3	14.8	12.3–17.7	14.8	12.0–15.8	20.0	17.7–22.5
≥ 50	14.3	11.9–17.1	21.4	18.8–24.2	11.3	9.3–13.7	11.9	10.0–14.1	19.6	16.9–22.6
Education (years)										
0-8	17.3	14.3–20.7	21.8	19.6–24.3	11.0	9.2–13.0	10.5	9.1–12.1	18.0	15.8–20.5
9-11	19.9	16.1–24.3	29.4	23.7–31.4	16.0	13.2–19.4	13.1	11.0–15.6	20.5	17.9–23.3
≥ 12 years	16.1	10.9–23.2	32.9	28.1–38.2	16.1	12.1–21.0	13.5	10.4–17.4	22.7	18.2–28.0
Economic classification^b^										
A/B	17.7	13.1–23.5	26.6	20.8–32.6	15.0	12.1–18.6	12.2	10.3–14.4	20.5	17.2–24.3
C	18.8	15.6–22.5	24.5	21.9–27.4	12.6	10.5–15.0	11.3	9.7–13.1	19.0	17.0–21.2
D/E	15.7	12.5–19.6	22.2	19.7–24.9	10.1	7.7–13.1	10.1	7.5–13.7	18.0	14.5–22.0

^a^ Percentages adjusted for sample weights and post-stratified according to age and gender.

^b^ According to the 2013 Brazilian Economic Classification Criterion of the Brazilian Association of Research Companies (www.abep.org).

During the multivariate analysis (Table 3), the consumption of drugs by self-medication showed a positive association for the female gender (PR 1.33; 95%CI 1.21–1.47), the 10 to 19 years (PR 2.03; 95%CI 1.66–2.47), 20 to 39 years (PR 3.46; 95%CI 2.94–4.08), 40 to 59 years (PR 2.68; 95%CI 2.26–3.17) and 60 or more years (PR 1.95; 95%CI 1.63–2.34) age groups, those residing in the North (PR 1.76; 95%CI 1.44–2.16), Northeast (PR 2.33; 95%CI 2.00–2.72) and Midwest (PR 1.74; 95%CI 1.49–2.04) regions, and those that have one (PR 1.17; 95%CI 1.06–1.29) and two or more chronic diseases (PR 1.33; 95%CI 1.18–1.50).

**Table 3:** Crude and adjusted prevalence ratios or the use of at least one drug by self-medication, according to demographic, socioeconomic aspects, health conditions indicators and access to health services indicators. PNAUM, Brazil, 2014. (N = 40,833)

Variable	Crude PR	95%CI	p	Adjusted PR	95%CI	p
Gender			< 0.001			< 0.001
Male	1			1		
Female	1.44	1.31–1.59		1.33	1.21–1.47	
Age group (years)			< 0.001			< 0.001
0-9	1			1		
10-19	1.89	1.54–2.33		2.03	1.66–2.47	
20-39	3.25	2.72–3.89		3.46	2.94–4.08	
40-59	2.68	2.22–3.21		2.68	2.26–3.17	
≥ 60	2.15	1.78–2.59		1.95	1.63–2.34	
Color			< 0.001			0.045
White	1			1		
Black	1.19	1.04–1.37		0.93	0.81–1.07	
Yellow	1.63	1.25–2.13		1.11	0.88–1.40	
Brown	1.11	1.00–1.23		0.90	0.82–1.00	
Indigenous	1.93	1.18–3.16		1.37	0.89–2.12	
Education (years)			< 0.001			
0-8	1					
9-11	1.37	1.17–1.43				
≥ 12	1.37	1.15–1.52				
Economic classification*			0.858			
D/E	1					
A/B	1.03	0.90–1.20				
C	1.03	0.93–1.13				
Region			< 0.001			0.001
North	1.56	1.27–1.92		1.76	1.44–2.16	
Northeast	2.10	1.79–2.44		2.33	2.00–2.72	
Southeast	1.12	0.92–1.36		1.18	0.97–1.42	
South	1			1		
Midwest	1.18	1.44–1.98		1.74	1.49–2.04	
No. chronic diseases			< 0.001			< 0.001
0	1			1		
1	1.33	1.20–1.48		1.17	1.06–1.29	
≥ 2	1.33	1.20–1.47		1.33	1.18–1.50	
Health insurance			0.708			
No	1					
Yes	1.03	0.90–1.18				
Hospitalized in the previous year			< 0.001			
No	1					
Yes	1.29	1.14–1.47				

* According to the 2013 Brazilian Economic Classification Criterion of the Brazilian Association of Research Companies (www.abep.org).

The respondents reported using 57,424 different kinds of drugs, 8,545 of which (17.0%) were characterized as being a self-medication type. It was not possible to identify the source of who recommended 3,258 medicines, these were therefore considered losses.

The drugs most commonly consumed, according to the first level from the ATC classification were those used for central nervous system (34.3%), followed by products used for the musculoskeletal apparatus, digestive tract and metabolism, respiratory system, genitourinary system and sex hormones, anti-infective for systemic use and others. In regards to the distribution of drugs by therapeutic group from the ATC classification (second level), the most frequent were: analgesics (33.4%; 95%CI 31.4–35.4), followed by the muscle relaxants and anti-inflammatory or antirheumatic agents, totaling 58.9% of medications consumed (Table 4).

**Table 4:** Distribution of drugs used in self-medication, according to the ATC classification (2nd level). PNAUM, Brazil, 2014. (N = 8,545)

Therapeutic subgroup	%	95%CI
Analgesics	33.4	31.4–35.4
Muscle relaxers	14.8	12.5–15.3
Anti-inflammatories and antirheumatics	11.7	10.4–13.2
Cough and cold remedies	5.9	5.0–6.9
Supplements (general nutrients)	3.9	3.3–4.6
Drugs for acid-imbalance-related disorders	3.8	3.3–4.6
Sex hormones and genital system modulators	3.1	2.5–3.7
Drugs for functional gastrointestinal disorders	2.8	2.3–3.3
Plants	2.7	2.2–3.4
Vitamins	2.4	1.8–3.1
Antibacterials for systemic use	2.3	1.9–2.8
Other	14.3	13.1–15.6
Total	100	

The drugs most frequently consumed by self-medication (Table 5) were: dipyrone, single fixed-dose caffeine-orphenadrine-dipyrone and paracetamol. Most of the medications were generally classified as non-prescription drugs (65.5%), followed by the sale of prescription medication (24.4%) and the special control drugs (0.5%) (Table 5). 48.5% of the 12 most used drugs (self-medication) were non-prescription drugs.

**Table 5:** Distribution of the 12 most commonly used drugs by self-medication, according to the ATC classification (5th level) and legal category. PNAUM, Brazil, 2014a. (N = 8,545)

Drug	%	95%CI	NPD^b^	PD^c^
Dipyrone	15.4	13.9–17.1	X	
Caffeine + orphenadrine + dipyrone	12.1	10.8–13.6	X	
Paracetamol	11.4	10.2–12.8	X	
Caffeine + carisoprodol + diclofenac + paracetamol	3.6	3.0–4.4		X
Diclofenac	3.5	2.7–4.5		X
Caffeine + dipyrone + isometheptene	3.3	2.5–4.2	X	
Ethinylestradiol + levonorgestrel	2.5	2.0–3.0		X
Ibuprofen	2.3	1.8–3.0	X	
Phenylephrine + chlorpheniramine + paracetamol	2.2	1.6–2.9	X	
Omeprazole	1.8	1.4–2.4		X
Caffeine + chlorpheniramine + dipyrone	1.8	1.4–2.5	X	
Nimesulide	1.6	1.2–2.1		X

^a^ Corresponds to 61.4% of the drugs used for self-medication.

^b^ Non-Prescription Drug.

^c^ Medication for sale with prescription or single-use prescription (red + black stripe).

## DISCUSSION

The prevalence of self-medication in Brazil was lower than that found by Carvalho et al.^4^ (2005), in a study in Brazil, and by Knopf and Grams^9^ (2013), in Germany. However, the prevalence was greater than that found in Sri Lanka^26^, Cuba^8^, and Spain^7^. We found variations in five Brazilian regions – the Northeast, Midwest and North, in which prevalences greater than the national prevalence were found.

Brazil is currently undergoing many transformations in healthcare, in which financial and infrastructure investment have been observed aimed at increasing the supply of health services, mainly in the area of primary care, with the Brazilian Family Health Strategy, and in the area of pharmaceutical care to ensure free access and rational use of medicines by professionals, and the community in general^17^ ^,^ ^23^. These transformations happen in different ways among the regions, however, despite the regional inequalities being exposed, greater access to medical services may promote less self-medication. Self-medication can also be considered to be within an acceptable range of magnitude^a^, because, as was noted during this study, the use of drugs for self-medication is restricted to self-limited acute diseases, such as stomach or intestinal problems, fever, pain, flu, colds or allergic rhinitis, nausea and vomiting, among others, and mostly to non-prescription products (65.5%). This result is in line with the results from a study performed in Brazil by Ahmed et al.^1^ (1997), in which the painful symptoms accounted for 24.3% of the motivations behind people practicing self-medication, with viral or infectious problems (high respiratory infection and diarrhea) accounting for 21.0%. Headache was found to be the main complaint for self-medication, followed by respiratory and digestive symptoms, during a study by Vilarino et al.^24^ A study by Carrera-Lasfuentes et al.^3^ found that pain was the most reported complaint.

No significant association was found between the practice of self-medication and economic classification. This result can be explained by the fact that the most frequently consumed drugs are of low cost, easy access and are most frequently prescribed^4^ ^,^ ^5^, including those free of charge medications made available from the Brazilian Unified Health System, such as those subsidized by the *Farmácia Popular* (Popular Pharmacy Program) (as is true for dipyrone, acetaminophen, and ibuprofen).

The finding that self-medication is most practiced by female individuals has also been found in other publications^2^ ^,^ ^4^ ^,^ ^9^, despite national and^11^ international^3^ ^,^ ^8^ ^,^ ^14^ ^,^ ^22^ ^,^ ^26^ studies indicating that self-medication is more prevalent among men. The results of this study may have influenced by the fact that women suffer more with headaches, muscle pain and chronic pain conditions, such as migraine, and use painkillers and muscle relaxers from an early age for pain relief during menstruation or dysmenorrhoea^2^ ^,^ ^19^.

Self-medication was also associated with the various age groups from the study, with the most prevalent users being between the ages of 20 and 39 years. This result can generally be attributed to health problem types that involve self-limited acute diseases, common to all ages, and the kind of medication consumed, with analgesics being the main therapeutic group used in the practice of self-medication^4^ ^,^ ^6^ ^,^ ^8^ ^,^ ^9^ ^,^ ^11^ ^,^ ^14^ ^,^ ^20^ ^-^ ^22^ ^,^ ^24^, irrespective of target population investigated and recall period.

This fact could also explain the positive association between the practice of self-medication with the various regions in Brazil and among individuals with chronic diseases or conditions, since analgesic/antipyretic medications are generally the second most used drug in Brazil and in the five regions of the country, second only to the antihypertensive drugs^5^.

In the case of the association of self-medication with age and the presence of chronic diseases, the results of this study differ from those found in Bambui, Minas Gerais^12^, where a negative association was found for age and self-medication showed no association with a history of disease or selected chronic conditions. This fact may be explained by the difference concerning when the research was conducted, since the emergence of new and old infectious diseases such as dengue fever, zika and chikungunya, the increasing prevalence of diseases arising from environmental degradation, environmental pollution and climate change^17^ may have intensified the appearance of self-limited acute diseases that encourage the practice of self-medication.

The most frequently consumed drugs were dipyrone, single fixed-dose dipyrone, orphenadrine and caffeine, and paracetamol. Similar data were found in national^1^ ^,^ ^24^ and international^8^ ^,^ ^11^ studies. These drugs are generally found in household medical supplies^10^ and are normally used to relieve signs and symptoms or acute^11^ ^,^ ^12^, minor or self-limited complaints.

The elevated use of painkillers for self-medication reflects the high prevalence of pain in the general population^3^, which is caused by tension, stressful situations or physical demand, thereby representing a damaging aspect in people’s quality of life. Abusing analgesics can lead to chronic headaches. A study performed in Colombia^19^ showed that the prevalence of headaches due to overuse of analgesics was 4.8%, which is higher than the prevalence in developed countries. On the other hand, the analgesic that relieves pain is the same one that is used to treat viral febrile, bacterial or inflammatory diseases. Nonsteroidal anti-inflammatory drugs (NSAIDS) are also attractive because they act in multiple ways: analgesic, antipyretic, and anti-inflammatory. Another aspect that favors and influences the consumption of these products is the advertising conveyed in the general media by pharmaceutical industry^16^.

Although the most drugs consumed are non-prescription drugs, it is important not to underestimate the possible toxic and adverse effects that might follow in their users. Concerning analgesics and (NSAIDS), some of the results worth citing are gastrointestinal disorders, allergic reactions and kidney effects^14^. During a the study by Martinez et al.^13^ (2014), the authors showed that practicing self-medication, among those interviewed, resulted in a rate for adverse effects equal to 15.1%; whereas a study by Paula, Bochner and Montilla^18^ (2012), which evaluated hospitalizations of the elderly due to intoxication and adverse reactions to drugs in Brazil, analgesics, antipyretics and non-opioid antirheumatics were responsible for 37.0% of hospitalizations by self-intoxication, occupying the fourth position in hospitalizations according to these situations, and were most related to cases of concussion.

In this study, the place from which the drugs used in the practice of self-medications were purchased was not analyzed, but it is likely that the most of them were purchased in pharmacies or drugstores or were leftover or from reserve stocks found in pharmacies, as was evidenced by at Laste and et al.^10^ (2012).

Other limitations of this study are related to the different recall periods employed to investigate the use of medications and the possibility of memory bias. This occurs most profoundly in the case of using any drugs in the 15 days preceding the interview, and also in information given by legal guardians of 15-year-olds with some cognitive disorder or inability to answer the questions. The use of some medications may also have underestimated while the study was ongoing, such as those that are commonly used, mainly in the South region of the country, to treat respiratory problems during winter.

In conclusion, the Brazilian population, with some regional differences, is no stranger to self-medication practice. This practice appears to be influenced by being female, residing in the Northeast, Midwest and North regions of Brazil, and by the presence of disease or chronic conditions. Most of the drugs consumed do not require prescription, but they are not risk-free, meaning that they deserve greater attention from managers and health professionals, as the possible intoxication and adverse effects may increase spending on health. Considering the aforementioned, regarding the practice of responsible self-medication, encouraged by the World Health Organization^a^, government investment must largely be used to promote sensible use of medication and include strategies for their use in the training of future health professionals; these aspects must be a continuous in nature or put in practice by the Brazilian Ministry of Health.

## Footnotes

a World Health Organization. The role of the pharmacist in self-care and self-medication. Report of the 4th WHO Consultive Group on the role of the pharmacist. The Hague: World Health Organization; 1998.

b Ministério da Saúde. Agência Nacional de Vigilância Sanitária. Resolução RDC nº 138, de 29 de maio de 2003. Dispõe sobre o enquadramento na categoria de venda de medicamentos. *Diario Oficial Uniao*. 6 jan 2004.

c Ministério da Saúde. Secretaria Nacional de Vigilância Sanitária. Portaria nº 344, de 12 de maio de 1998. Aprova o regulamento técnico sobre substâncias e medicamentos sujeitos a controle especial. *Diario Oficial Uniao*. 1 fev 1999.
